# Study protocol: cost-effectiveness of multidisciplinary nutritional support for undernutrition in older adults in nursing home and home-care: cluster randomized controlled trial

**DOI:** 10.1186/1475-2891-13-86

**Published:** 2014-08-28

**Authors:** Anne Marie Beck, Annette Gøgsig Christensen, Birthe Stenbæk Hansen, Signe Damsbo-Svendsen, Tina Kreinfeldt Skovgaard Møller, Eigil Boll Hansen, Hans Keiding

**Affiliations:** EFFECT, The Nordic Kitchen, University Hospital Herlev, Herlev Ringvej 75, DK-2730 Herlev, Denmark; The Municipality of Frederiksberg, Stockflethsvej 4, DK-2000 Frederiksberg, Denmark; Danish Institute for Local and Regional Government Research, Købmagergade 22, DK-1150 Copenhagen K, Denmark; Department of Economics, Faculty of Social Sciences, University of Copenhagen, Øster Farimagsgade 5, Building 26, DK-1353 Copenhagen K, Denmark

**Keywords:** Undernutrition, Nursing home, Home-care, Quality of life, Multidisciplinary nutritional support

## Abstract

**Background:**

Older adults in nursing home and home-care are a particularly high-risk population for weight loss or poor nutrition. One negative consequence of undernutrition is increased health care costs. Several potentially modifiable nutritional risk factors increase the likelihood of weight loss or poor nutrition. Hence a structured and multidisciplinary approach, focusing on the nutritional risk factors and involving e.g. dieticians, occupational therapists, and physiotherapist, may be necessary to achieve benefits. Up till now a few studies have been done evaluating the cost-effectiveness of nutritional support among undernourished older adults and none of these have used such a multidisciplinary approach.

**Methods:**

An 11 week cluster randomized trial to assess the cost-effectiveness of multidisciplinary nutritional support for undernutrition in older adults in nursing home and home-care, identified by screening with the Eating validation Scheme. Before start of the study there will be performed a train-the-trainer intervention involving educated nutrition coordinators.

In addition to the nutrition coordinator, the participants assigned to the intervention group strategy will receive multidisciplinary nutrition support. Focus will be on treatment of the potentially modifiable nutritional risk factors identified by screening, by involving physiotherapist, registered dietician, and occupational therapist, as relevant and independent of the municipality’s ordinary assessment and referral system.

The primary outcome parameter will be change in quality of life (by means of Euroquol-5D-3L). Secondary outcomes will be: physical performance (chair stand), nutritional status (weight, Body Mass Index and hand-grip strength), oral care, fall incidents, hospital admissions, rehabilitation stay, moving to nursing homes (for participants from home-care), use of social services and mortality.

An economic evaluation will be conducted to evaluate the cost-effectiveness of the multidisciplinary support.

Furthermore, interviews with nursing home and home-care management, nursing staff and nutrition coordinators in both the control and intervention groups, participants in the intervention group and the involved multidisciplinary team will be performed.

**Conclusion:**

In this study we will evaluate in a randomized controlled trial whether multidisciplinary nutritional support is cost-effective, in undernourished older adults in home-care and nursing home and contribute to important research.

**Trial registration:**

ClinicalTrials.gov 2013 NCT01873456.

## Background

Older adults in nursing home and home-care are a particularly high-risk population for weight loss or poor nutrition [1]. In Denmark as many as 50% of older adults in nursing homes suffer from unintended weight loss and, app. 20% of the residents and 12% of the clients have a body mass index (BMI) below 18.5 [2, 3].

The negative consequences of undernutrition, i.e. increased risk for morbidity and mortality, impaired cognitive, physical, and social function and hence reduced quality of life, and increased health care costs, hospital stays, more general practitioner visits, more intensive nursing care, increased requirement of nursing home-care etc. [1]. The increased need for care and social services leads to costs of billions of euro’s every year [1]. A Dutch study showed extra cost for managing nursing home residents at risk of undernutrition at 8.000 euro per nursing home resident and 10.000 Euro for undernourished residents [4]. And a Danish, Swedish and a Norwegian study have shown that older adults in respectively, nursing homes and home-care, who are undernourished, need more assistance with Activities of Daily Living (ADL) than older adults who are in good nutritional status and due to that, may add to the substantial and costly burden of care [3, 5, 6].

Several potentially modifiable nutritional risk factors increase the likelihood of weight loss or poor nutrition [7, 8]. Even though there is increasing evidence that the use of oral nutritional support (ONS) among nursing home residents improves weight and reduces mortality [9], the evidence for a benefit among older adults in home-care is very limited [9]. In addition, a much more structured and multidisciplinary approach, focusing on the significant modifiable nutritional risk factors and involving e.g. dieticians, occupational therapists, physiotherapist, may achieve additional benefits. Recently, we therefore developed and validated a nutritional risk screening tool, Eating Validation Scheme (EVS), which is designed for use among nursing home residents and home-care clients and includes eating habits, recent weight loss and the potentially modifiable nutritional risk factors, eating dependency, leaves 25% or more of food uneaten at most meals, chewing and swallowing problems with the aim of using these information in a multidisciplinary approach as needed [10]. The plan is to implement EVS all over Denmark. However, the EVS has only been tested in a small unpublished pilot study, including a train-the-trainer intervention and nutrition coordinators. However the results need to be confirmed by a proper randomized controlled trial, where the benefits of a multidisciplinary nutritional intervention aimed at residents and clients, who are identified by means of EVS, are assessed.

According to a recent systematic review, there is actually published some such multidisciplinary intervention among undernourished adults [11]. Unfortunately the 15 studies included in the systematic review had reported very few relevant outcomes and it was therefore not possible to conclude if multidisciplinary interventions were effective [11]. Only two of the studies included in the systematic review were performed among nursing home residents (and none among home-care clients). One of these actually documented a positive effect nutritional and functional status of a multidisciplinary intervention consisting of energy- and protein dense home-made oral supplements, exercise and oral care [12]. While the other found that a multidisciplinary intervention consisting of education of nutrition coordinators including train-the-trainer sessions were able to maintain nutritional status [13].

Up till now a few studies have been done evaluating the cost-effectiveness of nutritional support among frail older adults. Recently these studies were summarized in a systematic review [14]. The authors concluded that the use of nutritional support, mainly in the form of ONS or enteral feeding nutrition in the management of undernutrition could be efficient from a health economic perspective [14]. Two of the studies included in the review were performed among nursing home residents and home-care clients. However, none of the studies included in the review used a multidisciplinary approach. It could be expected that such a multidisciplinary approach would be accompanied by higher health care costs than usual care, due to the additional staff and assistance of physiotherapist, registered dietician, occupational therapist, etc. But it could also be expected that these higher costs are negligible compared with increased quality of life and the cost-savings this can potentially generate.

The aim of this study is to assess the cost-effectiveness of multidisciplinary nutritional support for undernutrition in older adults in nursing home and home-care.

## Methods

### Design

This study is designed as an 11 week cluster randomized controlled trial assessing the benefits of a new model for multidisciplinary nutritional support. The protocol has been send to the Danish Ethical Board which has concluded that approval is not needed and that the project can be carried on as described.

Older adults are eligible for this study when they are 65+ years old and achieve 2 points in the EVS (see below) [10].

The primary outcome parameter will be change in quality of life (by means of Euroquol-5D-3L, see below). Secondary outcomes will be: physical performance (chair stand), nutritional status (weight, BMI and hand-grip strength), oral care, fall incidents, hospital admissions, rehabilitation stay, moving to nursing homes (for participants from home-care), use of social services and mortality.

An economic evaluation will be conducted alongside the randomized trial to evaluate the cost-effectiveness of the multidisciplinary support.

Furthermore, interviews with nursing home and home-care management, nursing staff and nutrition coordinators in both the control and intervention groups, participants in the intervention group and the involved multidisciplinary team will be performed.

### Feasibility of recruitment and sample size

Unpublished results have shown that around 25% of nursing home residents will achieve 2 points in the EVS. According to our knowledge no nutritional support studies among nursing home residents and home-care clients have used Euroquol-5D-3L. However in a study of multidisciplinary nutritional support among another frail group of older adults, patients with hip fractures, Hoekstra and co-workers [15] used this measure and found a significant difference between intervention and control group in the Euroquol-5D-3L follow-up score of 0.145 (p = 0.004). Hence, with a statistical significance level of 0.05 and a power of 80% two groups of 65 older adults is calculated to be sufficient. The above mentioned unpublished study found that it was possible to screen 600 nursing home residents during an 8 weeks period.

Taking into account an expected loss to follow up of 10% during the 11 weeks of intervention based on a former multidisciplinary study among nursing home residents [12], and estimating equal findings among home-care clients, we aim to include a total of 145 older adults with 2 points in EVS. We therefore need to screen about 600. This number could probably be reached in approximately 8 weeks.

### Randomization

To avoid contamination from the intervention the participants will be randomized in clusters (based on [16]). The clusters consist of the participating nursing homes (three clusters) and home care areas (three clusters). Due to the limited knowledge about the benefit of nutritional support among home-care clients the aim is to assign randomly two of the home-care clusters to the intervention group. Participants, involved Research Assistants (AGC, BSHA, SD-S, and TKSM) and the Primary Investigator (AB) are not blinded for the intervention. Before starting the analysis the primary investigator (AB) will be re-blinded for participants’ group assignment.

### Population, inclusion and exclusion criteria

Older adults (65+ years of age), receiving home-care or living in one of two nursing homes living in the Municipality of Frederiksberg, screened with EVS by the nursing staff caregivers and, according to the staff care givers are able to complete the planned tests.

Participants will be excluded from the study when they are not able or willing to give informed consent.

### Nutritional status

Participants are eligible for this study if they are identified with 2 points according to EVS [9]. EVS contains information about eating habits, recent unintended weight loss, and the presence or absence of potentially modifiable nutritional risk factors (eating dependency, chewing and swallowing problems, acute disease, or acute change in chronic disease). The information is combined to give a total number of points, 0 point (no risk), 1 point (at risk), and 2 points (intervention).

### Control group

Before start of the intervention a nutrition education program will be performed educating selected staff members from the participating home-care and nursing homes to take the role as nutrition coordinator. The education of the nutrition coordinator includes three whole-day courses plus train-the-trainer sessions with other staff members (based on [13]) and local study circles in-between (based on [17]). These nutrition coordinators will be active in both the control and the intervention group. Also, in both groups standard interventions from physiotherapist, registered dietician and occupational therapist requested through the municipality’s normal assessment and referral system will be maintained.

### Intervention group

**Figure 1:**
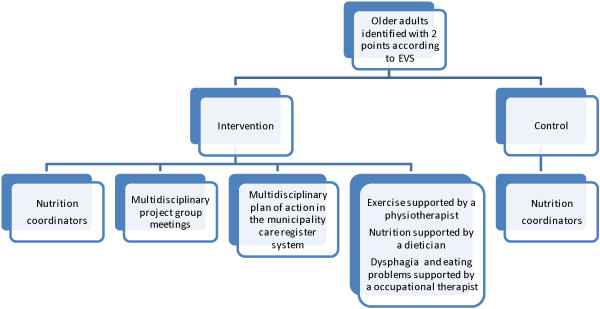
**The new model for multidisciplinary nutrition support.**

In addition to the educated nutrition coordinator, the participants assigned to the intervention group strategy will receive the new model for multidisciplinary nutrition support during 11 weeks. Focus will be on treatment of the potentially modifiable nutritional risk factors identified by screening with the EVS, by involving physiotherapist, registered dietician, and occupational therapist, as relevant according to the screening and independent of the municipality’s ordinary assessment and referral system. The intervention is coordinated by the Principal Investigator (AB) and the four Research Assistants (AGC, BSHA, SD-S, and TKSM) and contains a formalized multidisciplinary collaboration including a meeting once a week to discuss, evaluate and adjust the multidisciplinary support of each of the participants (see Figure 1).

#### Physiotherapist intervention

All participants in the intervention group will be offered to attend 30 to 45 min exercise program of moderate intensity twice a week. Focus will be on strength and balance exercises (based on evidence from [12, 18]) supervised by physiotherapists affiliated to the study and with regular attendance by the caregivers. The program will be individualized according to the baseline assessment and performed in groups of five participants, or if not possible, individually in the participants’ own home. The program will start with a warm-up session with activities that involved large muscle groups. This will be followed by functional strength exercise of the upper and lower extremities and progressive dynamic balance training. The level of exercise will be evaluated regularly to ensure that the same relative training intensity will be maintained in terms of load, number of repetitions, exercise duration, and degree of difficulty in balance training.

The intervention group will receive one bottle (125 mL) of an oral “training” supplement immediately after the two weekly exercise bouts. The oral “training” supplement will provide an average of 1010 kJ and 14.4 g of protein per 100 mL twice a week and there will be different flavors to choose from. If a participant do not attend the group exercise, when possible the supplement will be offered anyway.

#### Registered dietician intervention

The registered dietician affiliated to the study will be asked to consult the participants in the intervention group with unintended weight loss according to the EVS or the weekly assessment of weight. To assess the dietary intake the registered dietitian will perform an interview with the participants and if necessary with help from the relatives or caregivers at the initial assessment. The nutritional support will be according to a treatment protocol and based on the official recommendations [19]. The registered dietician will give the participants advice on how to raise the nutritional intake (i.e. consuming more dairy and whole-milk products, using butter and cream etc.), and discuss the importance of nutrition with participant, care givers and relatives (if possible). ONS and vitamin D and Calcium supplementation will be recommended as needed. The information will be supported by leaflets and an individual treatment plan made in collaboration with the participant.

The individual follow-up will be, in participant’s home, in the context of group exercise or by telephone. In addition, contact will be made to the caregivers, food service supplier, general practitioner, and others where appropriate. The time used for the initial assessment and follow-up will be documented.

#### Occupational therapist intervention

The occupational therapist affiliated with the study will be asked to consult the participants who suffer from eating dependency or chewing and swallowing problems according to the EVS. The task of the occupational therapist is to determine if the participant actually has swallowing or chewing problems, or if any help during meals is needed, and initiate intervention to solve these problems.

Specifically, the initial dysphagia assessment will consist of indirect and a direct swallowing tests. Indirect swallowing test assess the ability to be awake and alert, sit up, have control of the head, coughing and clear throat at request, swallowing own saliva and have a clear voice. Direct swallowing test consist of 3 steps. Step one: swallowing 3× teaspoon of water. Step two: Drinking 50 ml of a glass of water. Step three: observation during a meal (based on [20, 21]). There will be follow-up as needed, in participant’s home, in connection with the group exercise or by telephone. In addition, contact with be made to caregivers, food service supplier, dental hygienist, the visitation for the referral of eating aids and others where appropriate. The time used for the initial assessment and follow-up will be documented.

#### Compliance with intervention strategies

The physiotherapists will document the consumption of ONS (recorded as 1, ¾, ½, or ¼ portion consumed).

After each exercise bout, the physiotherapist will record each participant’s attendance, training intensity, and potential adverse events. The registered dietician and occupational therapist will document number of visits, reasons for cancelling appointments and possible problems with the suggested intervention strategies.

### Procedure for baseline assessment

After obtaining participants’ informed consent an inventory will be made of possible confounders. This includes the following baseline characteristics:

- Socio-demographic data (age, gender, living conditions, i.e. in a nursing home or in own home)
- Social services, i.e. hours and type of home help and home nursing (for home-care clients) from the municipality care register system.
- Functional, nutritional, medical, cognitive, psychological and social status by means of the Resident Assessment Instrument (RAI) for, respectively home-care (RAI-HC version 2.0) and nursing home (RAI-NH version 2.0). RAI is a comprehensive and standardized assessments system. Each item of the RAI has its own explicit definition and coding conventions, and a manual developed for the 2.0 versions describes how to ask questions, what to observe, and whom to contact for information. Both RAI-NH and RAI-HC has been translated into Danish (and several other languages). Studies have shown that inter-rater reliabilities for respectively the RAI-NH version 2.0 and RAI-HC version 2.0 items are adequate for research purposes, and both methods have been used in former Danish studies (see e.g. [5, 12]).

Trained nurses involved in the former studies above and affiliated with the present study will assess participant’s performance. All collected information will be discussed and crosschecked with the attending care givers and medical records. These data will also be collected at the end of the intervention period (see outcome parameters below).

### Outcome parameters

We will compare changes in quality of life, physical performance, nutritional status, oral care, fall incidents, hospital admissions, rehabilitation stay, moving to nursing homes (for home-care participants), use of social services and mortality between the intervention and control group.

All outcome parameters will be measured after inclusion (t = 0) and after 11 weeks (t = 11). In addition, the intervention group will have their weight measured approximately once a week. Primary outcome is health-related quality of life determined as EuroQol-5D-3L. If nothing else is stated data is gathered by the research assistants or the caregivers. All outcome parameters that will be measured are listed below.

#### Quality of life by means of EuroQol-5D-3L (t = 0 and t = 11)

EuroQol-5D-3L (EQ-5D-3L) is a standardized instrument for use as a measure of health outcome. The EQ-5D-3L descriptive system comprises the following 5 dimensions (5D): mobility, self-care, usual activities, pain/discomfort and anxiety/depression. Data will be collected by nurses affiliated with the study.

Each dimension has 3 levels (3L): no problems, some problems, extreme problems. The raw score must be converted to an EQ-5D-3L score ranging from 1.000 to -0.624 [22]. The EQ-5D-3L scores will be used to calculate utilities using the Danish tariff [23].

#### Physical performance by means of 30 seconds chair-stand (t = 0 and t = 11)

Participants are asked to fold their arms across their chest and to stand up and sit down on a chair without pushing off with arms, as many times as possible during 30 seconds. The arms may be used for assistance or for safety if need [24]. The mode of chair stand will be registered.

#### Nutritional status by means of weight, height, BMI and hand-grip strength (t = 0 and t = 11)

Weight (in kg to the nearest decimal) is measured (with participants wearing light indoor clothes, i.e. night dress) on calibrated weights present in the different settings. In addition, the participants in the intervention group will be weighed once a week before the start of the exercise and/or in relation to the registered dietician consultations. As measurement of height is often not feasible in this old and frail population with chronic disease, data about height will be retrieved from self-reported height. BMI is calculated as actual weight in kilograms divided by the square of height in meters.

Hand-grip strength will be measured (in kg) with a Jamar 5030J1 Hydraulic Hand Dynanometer. Participants will be seated with forearms rested on the arms of the chair. They are asked to perform three maximum force trials with their dominant hand and using the second handle position. The maximal grip score from the three values will be used.

#### Oral care by means of RAI-NH, RAI-HC and observation (t = 0 and t = 11)

The RAI-NH version 2.0 and RAI-HC version 2.0 contain information about oral care. These data will be supplied by grading the hygienic level based on three pictures of dental plaque: no plaque, plaque covers less than half of the tooth surface, and plaque covers more than half of the tooth surface.

Pictures are provided from a public dental health care program in the municipality of Aarhus.

#### Fall incidents, hospital admissions, rehabilitation stay, moving to nursing homes, and mortality (t = 11)

These information’s will be gathered by means of data from the RAI-NH version 2.0 and RAI-HC version 2.0 assessments and the municipality care register system.

For each participant the same trained nurse will collect RAI-NH version 2.0 and RAI-HC version 2.0 data at t = 0 and t = 11.

#### Economic evaluation (t = 11)

At the end of the trial, having received the data, an economic evaluation of the intervention will be carried out. This evaluation will take the form of a cost-effectiveness study, in which the intervention with its different forms of nutritional support is modeled as a decision tree, so that the citizen is followed through the intervention, costs and effects being assigned to its different varieties.

The effects of the intervention will be measured both in terms of changed body weight and as change in health-related quality of life, measured in QALYs on the basis of the EQ-5D-3L measurements. Time used for the different tasks will be registered and used in the calculations. The cost of the intervention includes cost of screening, cost of the multidisciplinary nutritional support and cost of training the staff for the specific tasks specified by the intervention. The results will be compared to other interventions in healthcare, and the uncertainties in the assessments will be illustrated by suitable sensitivity analyses.

#### Interviews (t = 0 and t = 11)

Before the intervention managers and selected staff members of the participating nursing homes and home-care districts will be interviewed on their attention to the nutritional state of the citizens, practice with respect to screening for nutritional problems, nutritional support, division of responsibilities and tasks, and inhibitory and promoting factors for nutritional support. At the end of the intervention managers and selected staff will be re-interviewed on the same issues as to assess whether practice has changed in the intervention clusters and the control clusters.

At the end of the intervention the multidisciplinary team will be interviewed on their performance of the intervention, the collaboration within the team, and the collaboration with nursing-home staff and home-care staff.

At the end of the intervention a selected small number of participants of the intervention will be interviewed on their expected benefits from participating, experienced benefits, and their assessment of the intervention.

### Organization

The Primary Investigator (AB) is overall responsible for the study. The Primary Investigator will be assisted by four Research Assistants (AGC, BSHA, SD-S, TKSM) who are responsible for the selection of the final participants’, the cooperation with nursing staff care givers and the multidisciplinary team, the data collection measurements and reports. Data flow will be controlled by the Primary Investigator. Data-entry and control will be conducted by the Research Assistants under supervision of the Primary Investigator. The Primary Investigator is responsible for the data cleaning and analysis. The Primary Investigator will also be assisted by HK and EBH who will be responsible for, respectively, the economic evaluation and the interviews, including the analysis of these data.

### Statistical analysis

All statistical analysis will be performed using SPSS for Windows. Data will be entered in EXCEL and will subsequently be exported into SPSS software for analysis. Data will be analyzed by the Primary Investigator who is blinded for the results of randomization. All participants will be included in the analysis, regardless of whether they have completed the study or not. Depending on the data type and distribution t-test, Mann-Whitney U test and Chi2 test will be used to compare changes within and between the groups.

## Discussion

When designing the study protocol we had to make a number of considerations, which may lead to the following strengths and weaknesses:

### Strengths

According to our knowledge this is the first prospective randomized controlled trial evaluating whether multidisciplinary nutritional support is cost-effective in a randomized design. This study is apparently also the first study of multidisciplinary nutritional support among home-care clients.

Both are highly relevant, respectively, for a research point of view and for the health economy aspect.

The methods used by the multidisciplinary team and most of the planned outcome measurements have been used in other studies and proven feasible among the target population for the present study. Also the RAI-NH version 2.0 and the RAI-HC version 2.0 has been used in former research studies among, respectively, Danish nursing home residents and home-care clients (e.g. [5, 12]). This gives us the opportunity to compare our results with the results of the former studies and is also why it has been decided to use these older versions of the Resident Instrument Assessment, instead of the recently developed newer versions.

We have chosen not to use strict exclusion criteria, even though they are suffering from a variety of chronic diseases and have different living conditions. Furthermore, the involved multidisciplinary team, registered dieticians, physiotherapist and occupational therapist are already today available in the Danish municipalities. Finally there is a lot of focus on rehabilitation which also involves a multidisciplinary approach – even though this is often without a nutritional support. All in all, this means that if the results of a broad study like this one are positive, it justifies wide implementation in the municipalities.

### Weaknesses

We have chosen to include participants with 2 points in EVS, instead of using e.g. the Mini Nutritional Assessment (MNA), which might limit the comparability with other studies. The reason for choosing EVS is that this tool, in contrast to e.g. the MNA is developed and validated in order to identify older adults in home-care and nursing home, who could benefit from nutritional support. However, whether this is actually true has never been examined before [10].

The participants included in the study should be able to complete the planned tests and to give informed consent. These criteria might exclude demented and functionally impaired persons and hence reduce the representativeness of our findings.

Furthermore, according to our knowledge, there have only been performed a few randomized controlled studies of nutritional support among home-care clients and none using a multidisciplinary approach.

Finally the EQ-5D-3L has apparently not been used in this population before. Hence our expectations about feasibility of recruitment and sample size assessment are based on relatively few published data, primarily among nursing home residents. However we expect that the home-care population is less frail than the nursing home population and therefore may be more willing to participate.

In relation to this, we have decided to use cluster randomization to avoid contamination. Hence the estimated sample may be too small to detect a significant difference [25]. Due to limited time and resources it is not possible to increase the number of clusters. To try to compensate for this we aim to include statistical analysis of the percentage of individuals in the different clusters that benefits.

We could have decided to use the Short Physical Performance Battery (SPPB) for the functional assessment. SPPB measures chair rise, balance and gait speed. However instead we decided to use methods which we had experience working with from former studies, and which gives some of the same information as the SPPB, i.e. 30 seconds chair-stand and RAI.

We have decided to use quality of life as our primary outcome. Another relevant outcome measure could have been ADL, especially since former studies have shown that older adults in respectively, nursing homes and home-care, who are undernourished, need more assistance with ADL than older adults who are in good nutritional status and due to that, may add to the substantial and costly burden of care [3, 5, 6]. Apparently it seems difficult to convey improvement in ADL to cost, since the price is unknown.

Also it could have been relevant to consider the cost of hospital admissions, visits of general practitioners, or moving to nursing homes. However our sample size lacks power in order to be able to detect a significant difference in these outcomes.

It could have been interesting to evaluate the co-morbidities of the participants since pathologies may interfere with food intake and nutritional status change. Unfortunately we do not have access to this information.

## Conclusion

In this study we will evaluate in a randomized controlled trial whether multidisciplinary nutritional support is cost-effective, in undernourished older adults in home-care and nursing home. According to our knowledge no other studies has assessed this ad hence the study will contribute to important research. If the expected higher costs are negligible compared with increased quality of life and the cost-savings this can potentially generate, the results might influence common practice positively.
